# Pro-lactation cesarean section: Immediate skin-to-skin contact and its influence on prolonged breastfeeding

**DOI:** 10.3389/fsoc.2022.908811

**Published:** 2022-09-27

**Authors:** José Octavio Zavala-Soto, Laritza Hernandez-Rivero, César Tapia-Fonllem

**Affiliations:** ^1^Programs of Master and Doctorate in Social Sciences, University of Sonora, Hermosillo, Mexico; ^2^Obstetrics Department of the San José Hospital of Hermosillo, Hermosillo, Sonora, Mexico; ^3^Obstetrics Department of the Noroeste Medical Center in Hermosillo, Hermosillo, Sonora, Mexico

**Keywords:** prolonged breastfeeding, cesarean section, skin-to-skin contact, exclusive breastfeeding, breastfeeding support, pro-lactation cesarean section

## Abstract

Mexico has a high rate of cesarean sections and one of the lowest prevalences of exclusive breastfeeding in all of Latin America. There are known factors that can compensate for the disadvantages and drawbacks of cesarean delivery over breastfeeding. In terms of studying the variations of breastfeeding experiences, this work specifically concentrates on exploring different changes in the technique of cesarean section, related to immediate Skin-to-Skin Contact for women with high and low risk pregnancies, which may in turn influence Maternal Satisfaction and the choice of Prolonged Breastfeeding. A convenience sample of (*n* = 150) women who underwent cesarean section in a private hospital in Mexico between the years 2015–2020 participated in this study, the participants answered a structured interview protocol designed for the specific purposes of this study. The analysis was guided grounded theory. The majority of these participants (*n* = 121, 82.3%) were in labor before entering a cesarean section. The most common indications for cesarean section were those of active-phase arrest and regarding maternal complications, previous cesarean sections (*n* = 59) and hypertensive complications (*n* = 15) were the most frequent. For fetal complications, non-cephalic fetal positions (*n* = 12) were reported as the most common. Despite the different conditions of their cesarean sections, almost all the women experienced Skin-to-Skin Contact during the cesarean section. Almost all of them managed to breastfeed for more than 6 months and many of them breastfed their babies for up to 2 years. The main factors associated to prolonged breastfeeding and satisfaction were higher education degrees, immediate skin-to-skin contact during surgery and counseling on breastfeeding after the baby was born. Our findings highlight the importance of considering adjustments during and after a cesarean section, making it more focused on women and toward better probabilities of achieving prolonged breastfeeding in Mexican women. This being a first step for future studies of direct interventions in the breastfeeding process, such as the management of skin-to-skin contact and professional support after birth for guided breastfeeding.

## Introduction

Factors influencing theincrease in cesarean sections include those associated with the mother, the physician, and the situational context. In Mexico, cesarean section rates in private hospitals are very high and the public policy of reducing mortality has resulted in an increase in the number of cesarean sections that also affects indigenous populations apparently due to the centralization of care in hospitals and overmedication (Freyermuth et al., 2017). From an anthropological approach, medicalization is often referred as a situation where the practices and knowledge of the medical institution extend its function toward control, where these are not only geared toward curative and preventive matters (Menéndez, 2003). This attempt to control birth, which culminates in the overflowing rate of surgical births, is considered the clearest and most frequent sign of medicalization of health in obstetrics (Campiglia, 2022). Especially since the excessive prescription of the need to complete a pregnancy by cesarean section, and even more so if a trial of labor was not attempted, is considered as one of the most frequent interventions in the field. Avoiding this hyper-medicalization of birth together with the validation of the sexual and reproductive rights of women and families are considered as the two pillars of the humanization of birth care (Castrillo, 2022).

Traditional cesarean section is considered as a surgical act in which the protagonists are the surgeons who capture all the attention and women are considered as patients who passively receive treatment and undergo a procedure. However, by choosing medications and techniques tailored to each laboring woman achieving a surgery without pain but also without side effects and with a holistic approach of the entire clinical team can make the birth experience more humane, positive, and special by not forgetting that it is a “birth” and not just a “surgery” (Nolan, 2009; Hung, 2011; Crenshaw et al., 2012; Capogna and Boer, 2017).

Given that humanization is not solely based on the type of delivery, it is essential that in both vaginal and cesarean births, awareness of women's fundamental rights is raised, and medical care is provided based on the best and most recent scientific evidence without allowing medical judgment to be clouded by secondary factors such as women's own or institutional needs. If a woman is placed as the main protagonist of obstetric care, taking into account her emotional needs as well as her physical ones, and is made to feel safe, confident, and cared for in an intimate and warm environment, genuine humanized care can be achieved and thus seeking the most positive maternal and child health outcomes (Bergeron, 2007; Kukla et al., 2009; Malacrida, 2014).

Mothers who have gone through a long labor in which it has been necessary to intervene in order to help her along the expulsive period (either due to maternal fatigue or the baby's difficulty in finishing descending through the birth canal), as well as the mothers who have had a cesarean section, may have to endure postpartum process with limited maternal movement, wound pain, or difficulty adopting proper positions to breastfeed their baby (Tully, 2014). Newborns can also be directly affected in their abilities to feed from their mother's breast by interventions carried out during childbirth. The use of narcotics for maternal analgesia may influence neonatal reflexes related to nursing. It has been observed that newborns birthed by cesarean section or instrumental delivery may have more problems with attachment and inadequate suction to the mother's breast (Lau et al., 2015; Hobbs et al., 2016). These are some of the reasons why a delay in the initiation of breastfeeding has been attributed to women who have had a cesarean section or an instrumental delivery (Chien, 2007; Stevens et al., 2014; Haghighi, 2015; Patel et al., 2015).

As an evidence-based practice related to these goals, Skin-to-skin contact (SSC) refers to the placement of the naked newborn on their mother's bare chest. The baby will face their mother with their head to the side and both of them will be covered by a blanket. The proven benefits of skin-to-skin contact between mother and baby have been the subject of interest of different Cochrane reviews in the last 15 years (Moore et al., 2007, 2012, 2016). After being found beneficial after vaginal delivery, it is now also recognized as a desirable situation during cesarean section. Regarding its effect on lactation, it is known that SSC has a direct relationship with higher rates of exclusive breastfeeding (Sharma, 2016a; Comité de Lactancia Materna de la Asociación Española de Pediatría, 2017; Schneider et al., 2017; Brimdyr et al., 2018). Newborns who experience skin-to-skin contact with their mother develop behaviors such as crawling to the breast (Debes et al., 2013) and more effective suckling (Cantrill et al., 2014), in addition to the opportunity to express the necessary attention demands that trigger neuropsychobiological pathways that activate maternal behaviors and a more immediate response to the needs of the infant with increased lactogenesis (Conde-Agudelo, 2016). According to Stevens et al. (2014), the ideal time to initiate skin-to-skin contact is within the first 30 min of the newborn's life, even during cesarean sections, in order to leave a mark on the programming of the future physiology and behavior of the dyad, given this may impact the effectiveness of breastfeeding.

These changes in the surgical technique have been called humanized cesarean section (after having gone through other names such as natural, mother-centered, or family-centered cesarean section) they have the characteristic of seeking to involve the woman in the birth of her baby more actively, empowering women in their own decision-making regarding breastfeeding (Wen et al., 2020), in addition to incorporating elements that scientific evidence has shown to be very important in the health and development of the newborn, such as the delayed cutting of the umbilical cord and skin-to-skin contact as soon as possible. It is known that SSC during cesarean section can reduce maternal stress, improve satisfaction with the surgical experience, increase the rate of early initiation of breastfeeding and exclusive breastfeeding at 6 months of age of the newborn as recommended by the World Health Organization (Stevens et al., 2014). The benefits of SSC during cesarean sections have even motivated the modernization of surgical birth protocols in different countries for its implementation, as is the case of the Spanish Association of Pediatrics [(Asociación Española de Pediatría (AEP), 2021)]. In Mexico, some studies have shown that skin-to-skin contact is possible and that it implies benefits in breastfeeding (Rosas-Herrera et al., 2019); It has also been found that maternal satisfaction increases after this positive experience for mothers, with no notable differences between women who had a vaginal delivery or a cesarean section (García, 2017); in larger studies that included mothers who presented some complication and healthy mothers in addition to vaginal and cesarean births, the positive impact of skin-to-skin contact on breastfeeding is again observed (García et al., 2017). There are few places in Mexico that carry out practices during vaginal birth that favor prolonged breastfeeding and even fewer after cesarean birth. In some places they practice SSC in low-risk patients but not in those women with complications or high-risk pregnancies.

This leads us to ask the following research questions: how is a pro-lactation cesarean section performed and what do the women who lived through this experience describe about the way they feed their babies?

Our study seeks to understand how it is possible to carry out small modifications to the surgical technique by including skin to skin contact, without this implying structural changes in facilities or greater human resources, in order to describe the betterment of the procedure centered on women, their satisfaction and considering the health of newborns. These modifications in the technique attempt to meet the physical needs of women while also paying special attention to their emotional safety and psychological needs during labor, birth of her baby, and her breastfeeding process.

We are specifically interested in exploring the feelings and level of satisfaction toward breastfeeding that women experience after a pro-lactation cesarean section (humanized cesarean section with skin to skin contact procedures), while also reporting the amount of time and manner in which they breastfed, taking into account their expectations and both maternal and neonatal complications.

## Methodology

### Design

Our qualitative study, using a grounded theory approach and analysis, seeks to understand women's satisfaction with their breastfeeding process and their children's feeding during the first 2 years of life after a pro-lactation cesarean section.

The present research seeks to study the experience over time in the different stages of motherhood from the first hours of birth and up to 2 years of life of the babies belonging to the participating mothers. Different data collection strategies were employed such as participant observation, field notes, survey, and semi-structured interview. Participant observation by the researchers in this study was key to understanding the nature of the phenomenon, maintaining objectivity in order to record and analyze observations through field notes, photographs, and videos. In addition to a detailed review of the literature, the analysis of this information was the basis for the development of the questionnaire and interview applied to the participants.

A questionnaire was applied to a convenience sample of 150 participants who underwent cesarean section using the technique described in the appendix and which we will call pro-breastfeeding. In addition, in-depth interviews were conducted with a subsample of extreme cases of the same participants, which included cases with both maternal and infant complications, and for which category saturation was reached with 36 of these face-to-face interviews conducted in the private offices of the researchers themselves and at the same time the participants' treating physicians. The interviews were recorded and transcribed for analysis. Theoretical coding and clustering have been used for the thorough and exhaustive delineation of this study.

### Participants

This study took place in a private hospital called “Hospital San José” located in the city of Hermosillo, Sonora (northwestern region of Mexico). The hospital has an area especially designed for the care of pregnant women that includes LPR (Labor-Partum-Recovery) rooms, operating rooms, and hospitalization independent of other types of patients. However, the type of care that predominated was the traditional biomedical model, which was characterized by the majority of women in labor controlled by medication, with a high number of cesarean sections, routine separation of babies who were taken to the nursery and very often fed for the first time with milk formula. The role of the researchers during the study period involved efforts to implement a holistic model of care that included support for physiologic labor, increasing the number of women in trial of labor, and changing attitudes in the team of nurses, pediatricians and anesthesiologists to support the cesarean section technique that was being implemented for the first time and is the subject of the present study. The participants in our study in the setting of a private hospital were of middle and high socioeconomic level with professional education for the most part. Where in contrast to our sample, it is reported that the socioeconomic level of pregnant women attending a public hospital in northeastern Mexico was medium-low and low, with a predominant education of primary or secondary schooling and only 5% have received professional education or training (Barrón-Garza et al., 2021).

We employed a convenience/purposive sampling method to recruit participants. The study sample consisted of 150 women (Adults ≥ 20 years, *M* = 32.5). The participants were women who underwent a pro-lactation cesarean section from January of 2015 to November 2020. All participants with a cesarean section were invited and then volunteered to partake in the study. Inclusion criteria were that women (1) who had pregnancies longer than 32 weeks (2) had a pro-lactation cesarean section; (3) had a complete clinical file (see Table 1).

**Table 1:** Sample characteristics (*N* = 150).

**Characteristics**	***N***	***%***
Age (Mean = 32.5)
Age <35 years old	108	72%
Age > 35 years old	42	28%
Weeks of gestation (Mean = 39.2)
?37 weeks	142	95%
<37 weeks	8	5%
Education attainment
High school	11	7.3%
Bachelor's degree	109	72.7%
Graduate degree	30	20%
Indications for cesarean section
Previous cesarean section	59	39.3%
Nullipara secondary arrest of labor	47	31.3%
Failure to progress in labor	16	10.6%
Non-cephalic positions	12	5.3%
Hypertensive complication	8	5.3%
Other complications	8	5.3%
Type of delivery
Cesarean section during Labor	124	82.6%
Scheduled cesarean section	15	10%
Emergency cesarean section	11	7.4%
Use of oxytocin during childbirth
Yes	38	25.3%
No	112	74.7%
Skin to skin contact
Yes	132	88%
No	18	12%

### Ethical considerations

The study was approved by the hospital's ethical committee (Comité de Ética del Hospital General del Estado de Sonora). The collection of data and its processing were subject to the Federal Law on the Protection of Personal Data Held by Private Parties, which in Mexico has the objective of regulating the right to informational self-determination and that is applicable to all persons in the country, in both public or private sector at the federal and state levels. It was made clear to the participants that their participation was completely voluntary, that they could withdraw their authorization at any time, and that their data would be completely confidential.

### Data collection

Medical care at delivery was provided by two of the researchers in this study, using the same surgical technique and support routine for the mother-baby dyad (skin to skin contact procedure, described in Supplementary material), which helped us establish a relationship of trust with participants and make them aware that the purpose of the study was to obtain information on their breastfeeding practices, as well as improving nursing and mother care routines in the hospital.

As a first stage of this grounded theory project, a pre-structured survey of 18 questions was designed and applied, including some open-ended questions and others with a multiple choice response option to a purposive sample of 150 participants *via* web using SurveyMonkey tool in order to assess the diversity in the level of satisfaction of mothers and the type of food provided by mothers to their babies, as well as the different perinatal characteristics in each pregnancy and possible interventions by the health care team. Jansen (2013) describes the role and usefulness of this type of questionnaires called “qualitative surveys” in qualitative research. Qualitative surveys analyze the diversity of member characteristics within a population. Such diversity can be predefined or developed through open coding.

In the case of our study the last questions of the survey also provided key demographic information. The information obtained was complemented with the review of clinical records, memos and field notes taken during participant observation, interviews and in the different cycles of data analysis.

Data was collected through face-to-face intensive interviews in the hospital's consulting rooms. Interviews were carried out by the doctors who performed the pro-lactation cesarean section. An intensive qualitative interview technique was chosen for this study as a device that would allow us to explore our main topic and fit the participant's experience given it's direct, shaped, and unrestricted character (Charmaz, 2006). The intensive interview guide was formulated specifically for its use in this study, it included eight semi-structured focused questions. Participants' demographic information was obtained *via* a brief questionnaire and medical records. Memos and field notes were taken during the interviews. The initial codes and interview questions are shown in Table 2.

**Table 2:** Interview guide and coded dimensions.

**Dimension**	**Themes**	**Example of questions**
Maternal socio-demographic factors	Age, Birth complications, Mother's education	What was your age when your baby was born?
		What is your current educational level?
		Medical records
Maternal care and birth services	Management of cesarean section, Immediate skin to skin contact after delivery, Information about lactation, Support from healthcare professionals and Spatial proximity (“rooming in”).	How was your baby born? Describe your personal experience with labor.
		Did you have skin-to-skin contact with your baby for at least 30 min in the first 2 h of life?
		Was your baby after birth and until discharge from the hospital in the same room as you?
		Did you receive help and useful information after your baby was born to better initiate breastfeeding?
Successful lactation	Early lactation, Exclusive lactation, Prolonged lactation, Breastfeeding duration, Level of satisfaction with their lactation process	How long were you able to breastfeed your baby?
		Did you feed your baby with formula already during the first month of life?
		Did you exclusively breastfeed your baby for the first 6 months of life?
		Regarding the way you fed your baby during the first 2 years of life, how do you feel?

A summary of the questions included in the survey and the interview is shown in Supplementary Tables 2, 3. For the interview, the reason for the interview and the objective of the study were thoroughly explained to the participants, all their questions were answered, and it was disclaimed that the interview would be recorded. The order of the questions was flexible to foster an optimal flow of conversation. The average duration of each interview was 50 min and participants were given the opportunity to have a copy of the transcribed interview.

### Data analysis

Traditional grounded theory (Charmaz, 2006) guided the analysis. This approach attempts to touch on the actions and events in the studied world by naming, developing, synthesizing, integrating, and organizing codes from large amounts of data in order to propose an analytic handle to develop symbolic ideas for interpreting each different segment of data. The codes in this study were classified according to the factors that promoted prolonged breastfeeding and mother's satisfaction toward their breastfeeding journey and their experience with childbirth.

Our analysis had four main phases: (1) Classic line-by-line coding from the original transcript of the intensive interviews at the hospital, identifying the properties of each emerging concept; (2) Focused coding for retrieving most frequent and significant codes and explaining larger segments of data into categories; (3) Theoretical coding, which allowed us to analyze possible relationships between the categories developed in focused coding. Here, theoretical coding works as a way to abstract the way some of the substantial codes relate to others, as to derive the hypotheses that could be assimilated into a theory (Thornberg, 2014). When coding full interview transcriptions, theoretical codes lend from to the previously collected focused codes. These codes help explicate the meaning in a given segment of data, creating clear and evident connections between the data and the codes. In addition to conceptualizing how substantive codes are related, theoretical codes also move an analytic story in a theoretical direction. In this regard, the method of data collection not only shapes the materials for the analysis, but also frames the obtained codes. Chiefly, this type of grounded theory coding seeks to unify ideas analytically keeping in mind what the possible theoretical meanings of the data and codes might be (Charmaz, 2006) and (4) Clustering as a technique to create a visual representation of the organization of codes and outlining the emerging theory obtained from theoretical coding procedure. This technique has been commonly adapted and used with grounded theory methods, mainly because clustering provides a direct chart or map of the data analysis. In this manner, clustering maps help assess relative importance of the codes within a cluster and the relationships between them. Clustering begins with one code or central idea (usually represented with circles) and then moves on to clustering relationships between codes, drawing spokes or arrows from one code to any other code where it subsumes to signify relationships found throughout a main transcript or source. A clustering map uses configurations of clusters to construct an image of how the codes fit together and relate to other categories (Clarke, 2003; Charmaz, 2006).

All through our analysis, the similarities, and differences between the answers of the participating mothers were examined to obtain comparisons among raw interview segments, emerging codes, and categories. Coding differences between the researchers were mainly tackled and by discussion and clarified upon consultation of the original transcript.

## Results

**Figure 1:**
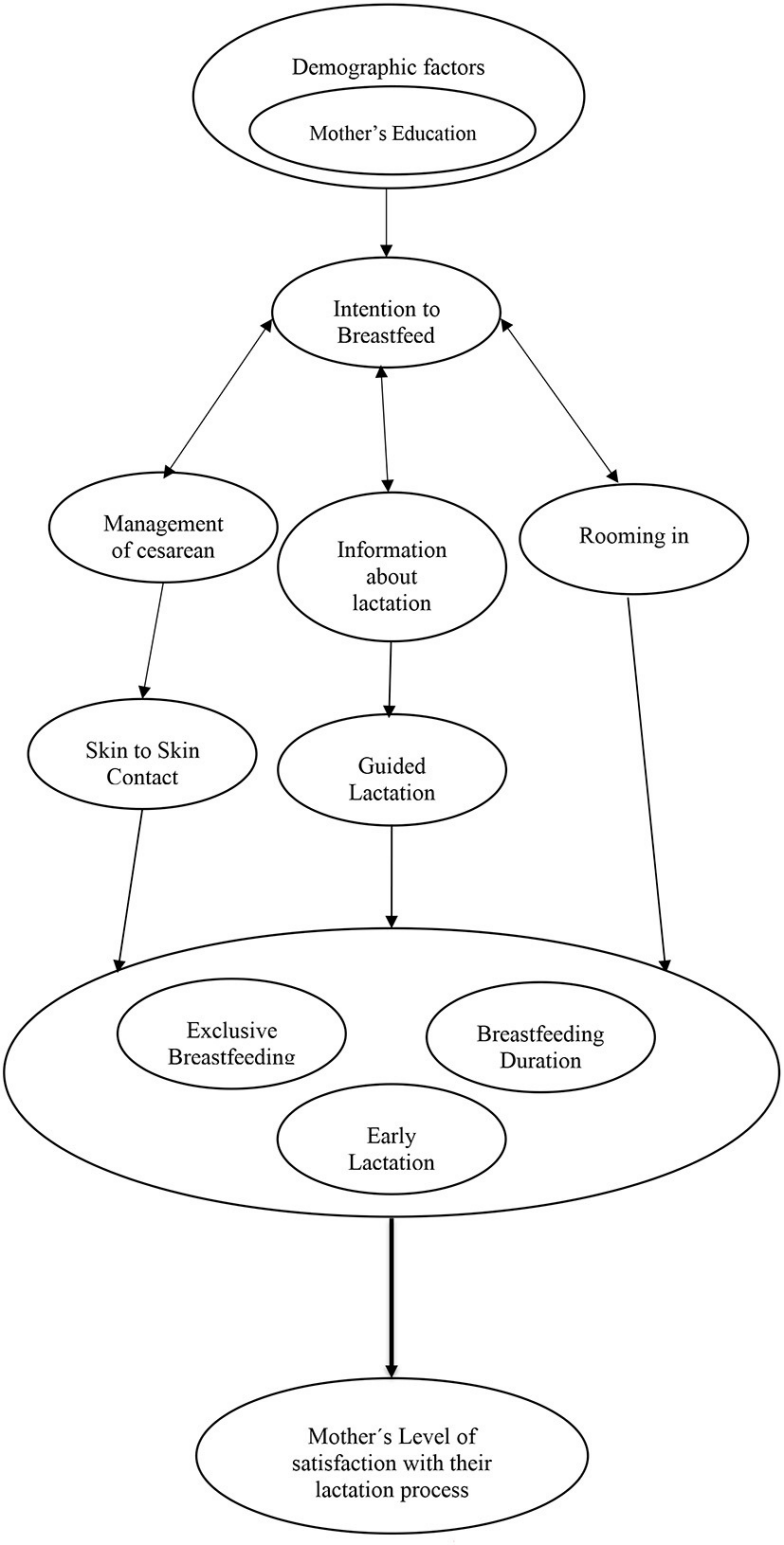
Clustering map of the grounded theory of successful breastfeeding after pro-lactation cesarean section.

Our analysis about women's satisfaction with their breastfeeding process and the main factors associated with it after a pro-lactation cesarean section is presented in two different manners. First, we focused on describing women's experience with breastfeeding from the first 2 h of their babies' lives up until the age of 24 months. In the study, three major themes emerged: maternal socio-demographic factors, Maternal care and birth services and Successful lactation. Second, we constructed a clustering map in order to construct an image of how the codes fit together and relate to other categories in the form of theoretical coding. The resultant themes and categories are displayed in Figure 1.

### Maternal socio-demographic factors

Some of the factors explored in this category were age (Min = 20 y/o, Max = 43 y/o, M = 32.5 y/o) maternal level of education, childbirth preparation and birth complications. The majority of the participants presented birth complications (Previous cesarean = 59, Nulliparous with secondary arrest at dilatation = 47, Failure of labor progression = 16, pelvic presentation = 12, hypertensive disorder = 8, Preterm births = 8, twin births = 3, and others such as uterine dehiscence, placental abruption, and eclampsia).

Most of the participants in this study group were considered high-risk because of a history of previous cesarean (39%), abnormal presentations, twins or premature babies, over-aggravated diseases such as hypertension and diabetes, or complications during labor such as women with their first pregnancy and whose labor failed to progress further (42%). However, because the conditions of mother and baby were stable, a pro-breastfeeding cesarean section was performed, which included emotional and physical support, warm environment, woman-centered care and mainly uninterrupted skin-to-skin contact with supervision of the first feeding at the breast. It was observed that most of these women breastfed exclusively for 6 months, including the twins and six of the eight premature babies (75%).

This sample includes 8 cases of true emergency cesarean section, including 2 participants who did not have skin-to-skin contact. One participant had seizures due to eclampsia at the end of pregnancy which required general anesthesia for surgery and therefore she was unable to breastfeed exclusively for 6 months. Another case of a participant with 32 weeks of pregnancy, diabetic, previous cesarean section, who presented complications with bleeding due to placental abruption, stands out. Her premature baby was transferred to Neonatal Intensive Care and the mother, with conviction and great determination to breastfeed, practiced stimulation and colostrum extraction, which after 24 h of separation she was able to start using to feed her baby. The participant reported having achieved exclusive breastfeeding for 6 months and continuing to breastfeed even after the baby was 2 years old. This case was an example of how maternal intention to breastfeed in a mother with a certain level of knowledge, attitude, intention, and excellent empowerment with a little help from the staff to improve her breastfeeding self-efficacy and overcome the great difficulties of known non-modifiable factors that affected her breastfeeding, thus achieving a successful and prolonged breastfeeding despite the extreme prematurity of her baby. A recent publication confirms the usefulness of maternal self-efficacy theory for the benefit of breastfeeding in preterm infants and that one should be aware of the role that cultural factors and institutional policies can play on it (Brockway et al., 2020).

A total of eleven infants in our sample required neonatal resuscitation at birth (7.5%) due to growth retardation, prematurity, respiratory distress, macrosomia, jaundice, and hypoglycemia. One of them had a congenital renal cyst and was later confirmed to have an autism spectrum disorder at 2 years of age. It is important to mention this because the literature reports a higher prevalence of autism spectrum and attention deficit problems among those born *via* cesarean section compared to those born vaginally (Zhang et al., 2019).

### Maternal care and birth services

The participants in the study expressed the importance of proper management of maternal care and birth services. The themes included in this category were management of cesarean section, immediate skin to skin contact after delivery, information about lactation, support from healthcare professionals and spatial proximity to their babies (“rooming in”).

The cesarean section technique was modified as described in the Supplementary material document, focusing mainly on making the participant feel accompanied, in an environment as relaxing as possible, with minimal discomfort during the procedure but aware of her surroundings and mainly trying not to separate her baby. One of the participants commented: “On the day of the birth, the entire medical team made me feel safe and calm, from the nurses to the anesthesiologist and the doctors behaved respectfully and kindly, making me the protagonist of the birth”.

The hospital protocols during the course of this study were changing and the nursing staff and professionals assisting in the mother's care were gradually accepting these changes, and in this case supporting the implementation of skin-to-skin contact between mother and baby during surgery and later in the recovery room. The cases in which skin-to-skin contact was not performed were because it was not allowed at that time in the hospital or because the conditions were not conducive to carry it out; there was only one case in which the mother preferred that the baby be taken to the nursery to rest after the surgery.

Most of the participants recognized that the labor test represented an important influence in preparing their bodies for breastfeeding to occur in a better way “In my case, when I breastfed him and he quickly sought it (her breast) and latched on, for me it was nice because you think about labor, about the hours that you endured were important, they were useful and he quickly sought my breast even though it was not natural (the birth) and physically you cannot see the milk but you know it is there and he was not given formula”. Labor and birth involve a complex and balanced interplay of hormones at such high levels as occurs with oxytocin in the mother that are not repeated at any other time in her life and whose alteration may have negative effects not yet well understood (Thul et al., 2020). Labor also provokes a different hormonal influence on the baby's body that allows it to adapt gradually, overcoming the physiological stress it represents and leaving it with different abilities to adapt to the air environment after birth, to feed itself, to stimulate its immune system and to have a better neurocognitive development and socialization with its environment (Hederlingova et al., 2017; Tribe et al., 2018; Bergman et al., 2019).

Accompaniment during labor by a trusted person or doula has a positive impact on women's satisfaction, physiological birth and breastfeeding rates (Rosen et al., 2008; Acquaye, 2021; Caponero et al., 2022). In our study, all participants were accompanied by a partner, family member or doula. One participant commented that a nurse had warned her that her husband would not enter the operating room because of hospital policies at that time: “I am very grateful that my husband was able to enter because it gave me great peace of mind to have his support and that he did not miss this important moment.”

One hundred and thirty two (88%) of the women participating in this study had immediate skin to skin contact with their babies during and after their cesarean section, claiming it as an important aspect of their breastfeeding journeys. One hundred and thirty five women reported spatial proximity was also a determinant factor. “My baby was always with me in the room” was one of the most frequent answers (63.3%) in this theme. Another recurrent theme was receiving information and support for lactation. Most of the women (*n* = 144, 96%) reported having received useful information after their baby was born to better initiate breastfeeding. In most cases the information was delivered by their physician (70%), a doula (16.7%) or a family member (4.7%). One of the respondents mentioned “The doctor's assistance did not end there, we received a lot of support in the hours and days following the cesarean section in terms of breastfeeding, which helped us a lot even until now to have exclusive breastfeeding as we wanted”. These results relate to those obtained by Chiou et al. (2014), where early skin-to-skin contact and rooming-in for more than 12 h were associated with increased chances for exclusive breastfeeding and breastfeeding at 6 months postpartum.

### Successful lactation

Most of the participants stated that they were seeking a vaginal birth and wanted to breastfeed their children. Although none of the participants achieved this goal by having a cesarean section, satisfaction with their childbirth experience in relation to their breastfeeding process was widespread in the sample studied. Expressions such as the following were very common “It was the most beautiful experience I have ever participated in, although I must say that it is exhausting and, in my case, it was a little frustrating that I could not have a natural birth, thank God both the baby and me are fine.”

Unmet expectations were related to the pain of contractions greater or less than expected, the duration of labor, and mainly to the desire for a vaginal birth. One participant who emphasized her frustration at not having achieved vaginal birth, reported that she “felt as if her own body had failed her.” The early and continuous rapprochement between the mother and her baby with the practice of skin to skin contact in the operating room was the theme most related to satisfaction and positive experience “It was a unique and very emotional moment, they put him on my chest, and we made skin to skin contact, the most impressive thing was that at that moment my baby sought my breast and began to suck”. This last category relates to findings obtained by Srivastava et al. (2014), where skin to skin contact interventions led to higher maternal satisfaction and better exclusive breastfeeding rates.

It was observed in the present study that nurses and staff responsible for the care of the newborn persisted in an attitude of control of the situation and on-call management that tends to separate the baby from the mother under multiple excuses such as bathing the baby, maternal rest, change of shift, among others, and to feed the baby with formula milk at the slightest suspicion that the baby is not eating regularly. The staff was not familiar with breastfeeding on demand, nor did they have the criteria to individualize the cases. This was the most difficult attitude for the researchers to modify in their role as participant observers, but perhaps it was also one of their main roles in counteracting these routines of the nursery staff and ensuring that a greater number of participants managed to remain in full rooming-in and exclusively breastfeed during their hospital stay, which has been described as having an impact on better breastfeeding rates when the woman is already at home and which mainly affects women who have undergone cesarean section (Bhandari et al., 2019).

Respondents quickly identified successful lactation, suggesting the significance of the event over time. 59% of the participants had already breastfed within the first hour and 84% had succeeded in breastfeeding within the first 4 h. 114 of the participants successfully breastfed their babies for more than 6 months after their cesarean section (6 months or more = 20%, more than a year = 27.3%, more than 2 years = 28.7%). Moreover, 104 of the women in this study practiced exclusive breastfeeding.

Regarding their level of satisfaction with their lactation process, most of these mothers reported high levels of satisfaction (*n* = 112, 74.7%), with mothers stating answers such as “I am pleased that I did the best I could”. Similarly, others (*n* = 16, 11%) reported lower, although good, levels of satisfaction such as “I feel good about it, but I would have liked to do some things differently,” which indicates that they probably did not reach their own goals, probably due to very high expectations regarding what each had considered successful breastfeeding.

## Conclusions

The present study helps us to represent breastfeeding as a complex phenomenon that can be influenced by different dimensions such as the woman's own characteristics, age, education, socioeconomic level, maternal intention to breastfeed, support from her immediate environment and, on the other hand, those that correspond to the birth environment and the quality of care received, such as the type of birth, orientation, and assertive support for breastfeeding. Our observations point out as the most influential factors for the success of breastfeeding in the study group the previous intention of the women to breastfeed, maternal care during birth which included not separating mother and baby, encouraging skin-to-skin contact and co-housing, and finally professional supervision of breastfeeding to overcome initial difficulties and achieve exclusivity of breast milk as the only food for the newborn.

Similar findings in literature corroborate that maternal intention to breastfeed prior to birth is a good predictor of the actual duration of breastfeeding but is influenced by the immediate support of family members and the medical team as well as the need to subsequently reinforce this intention to continue breastfeeding as a counterbalance to the social acceptability often against long-term breastfeeding (Rempel, 2004).

As shown in Figure 1, to achieve an early, exclusive, and prolonged breastfeeding, the variables that seem to stand out in the present study begin with the mothers' adequate knowledge about breastfeeding, which will condition their intention to breastfeed. This is followed by maternal care that favors skin-to-skin contact and no mother-baby separation, and finally, adequate breastfeeding counseling by professionals. Parental education and skin-to-skin contact are interventions already described in previous reviews (Beake et al., 2017) and we agree on the need to better understand the impact of cesarean section on maternal psychological recovery, breastfeeding physiology and infant feeding behaviors. Professional breastfeeding feedback and follow-up was important for participants with or without complications during pregnancy and birth, it was also common to need support or reinforcement when pain or perceived insufficient milk production arose at home. This adequate supervision and guidance from the certified counselor was related, in our perspective, to an improvement in maternal self-efficacy to breastfeed, eventually achieving correct technique and thus prolonged breastfeeding. As has been shown in other studies these interventions by certified specialists have impact even in situations of over-aggravated pathology and established breastfeeding problems (Chetwynd et al., 2019; Haase et al., 2019; Griffin et al., 2022).

Figure 1 also shows maternal satisfaction with breastfeeding beyond what was considered the success of breastfeeding by grouping early initiation and exclusive and/or prolonged breastfeeding. This is because we recognized satisfaction as a complex and multidimensional construct that can be influenced by individual factors, expectations, family environment (de Senna et al., 2020) and also in our study by complications with pregnancy or birth. Thus, we observed mothers who were satisfied with their efforts, even if the type of feeding was combined, and mothers who reproached themselves for not having been able to prolong an already very adequate exclusive breastfeeding. We recognize that the emotional health and state of mind of mothers influences the way they feed their babies (Gila-Díaz et al., 2020) and that it is a variable that should be taken more into account since the greater the maternal satisfaction, the better the breastfeeding rates, although there is a need for inclusion of mothers who by necessity or choice are supplementing with artificial formulas and who are not necessarily less satisfied for that reason alone.

The rate of cesarean sections, mainly and notably in the private setting in Mexico, is one of the most frequent examples of excessive intervention resulting from the induction of fear in the doctor-patient relationship and currently considered by some as obstetric violence (Espinoza, 2022). The main characteristic of the participants in this study group is that thanks to having received adequate and impartial information regarding the particular situation of each one during pregnancy, they all decided to attempt labor and cesarean sections were duly justified during this attempt. Even when the evolution of labor is not ideal or complications arise, a clear and simple explanation with easy to understand words and without the intention of inducing fear, involves and empowers the mother, while exercising her autonomy by making decisions together with the physician to continue with the next best treatment option, which may be a well-justified cesarean section carried out within high ethical standards that respond to humanization.

Policies for the humanization of obstetric care should be based on the recognition and guarantee of the rights of pregnant women and newborns, but also on the revision of routine medical practices. An exaggerated maternal risk approach used to induce fear and indicate a cesarean section due to the history of previous cesarean section would not have allowed the present investigation, given the high number of participants with previous cesarean section, who were well informed and attempting a vaginal delivery after having already experienced a cesarean section. Major medical organizations in obstetrics and gynecology in countries such as the USA, Canada, England, France and Australia support performing the “Trial Of Labor After Cesarean” because of the benefits that outweigh the risks in the vast majority of women who do not have an absolute contraindication (American College of Obstetricians and Gynecologists, 2010, 2019; Sentilhes et al., 2013; Gupta et al., 2015; Hauck, 2015; Dy et al., 2019; Queensland Clinical Guidelines, 2020).

Each woman should be able to make an adequately informed decision by assessing her individual interests and characteristics without being persuaded only by the preference of the attending health professional (Bernstein et al., 2012; Sindiani et al., 2020; Luo et al., 2021). This requires women to be properly informed and to have partners or family members, doulas, and healthcare professionals to support and care for them after their delivery (Basile et al., 2021). If the potential short- and long-term risks and benefits of childbirth are taken into account and explained in an understandable and deprofessionalized manner to the expectant mother, she will then be able to make informed decisions that may impact her satisfaction as observed in the study group.

Moreover, evidence continues to demonstrate the overall benefits of early initiation of breastfeeding, along with it reducing neonatal morbidity and mortality (Khan et al., 2015; NEOVITA Study Group, 2016; Sharma, 2016b). For newborns, early nursing with maternal colostrum is a consistent and significant predictor of prolonged exclusive breastfeeding (Patel et al., 2015; NEOVITA Study Group, 2016).

In current times, cesarean birth is recognized as a factor that directly affects breastfeeding by different mechanisms. In some communities of Mexico, it has been shown that cesarean births are associated with a later initiation of the first feeding to the mother's breast, shorter total duration of breastfeeding and greater use of artificial milk formula when compared with women who had a vaginal birth (Veile and Kramer, 2015).

In Mexico, modifiable factors associated with the abandonment of breastfeeding prevail, particularly in the early puerperium, such as feeding the babies other liquids other than breast milk during the hospital stay, using pacifiers when experiencing pain or discomfort in the breasts or nipples, or the belief of having to give powdered milk when the baby is not satisfied. As we have seen here, a greater culture of health professionals regarding breastfeeding is indeed necessary in order to modify these factors with simple interventions such as providing advice and personal and assertive support to mothers during their hospital stay, rooming-in and in the immediate postpartum period (Vázquez-Osorio et al., 2022).

This work suffers from a number of limitations—notably related to the selection of a private institution as the main scenario for our research. Hospital San José in Hermosillo, Sonora, is considered a specialty medical center, which has a modern infrastructure, high quality medical equipment and medical personnel specialized in women's health and wellness. Portraying a setting different than many other Hospitals and public health services in the same city. Therefore, It may not be appropriate to extrapolate our findings to other works carried out in the context of public hospitals in Mexico, the experience described by this sample are limited and cannot be generalized or claimed to be universal regarding maternity health assistance in Mexico

Furthermore, this work is also limited by its consideration of how women participating in this research were interviewed by the same personnel who had medically assisted them during their childbirth process. Although every women were voluntarily partaking in this sample and written consent was granted, the potential biases and conditioning of women's responses, deriving from the dual role of caregiver and researcher should be taken into account. Future directions are related to the improvement of the interview methods and assessing interviewer bias.

One of the main strengths of our work lies in highlighting emergent theory where it may be possible to carry out a cesarean birth care protocol focused on the emotional and psychological needs of women in which accompaniment is provided by a person of her choice, a calm and warm environment is sought, a delayed cutting of the umbilical cord in a timely manner and immediate and uninterrupted skin-to-skin contact between the mother and her baby is implemented. when, in addition to this, the adequate initiation of breastfeeding is supported, supervised, and guided by healthcare professionals, it is likely that, despite the maternal or neonatal complications that led to/come with cesarean section, better rates of prolonged breastfeeding will be achieved.

This study exemplifies how in the current paradigm of childbirth it is possible to unite one of the most common interventions of our time, cesarean section, with the holistic model of woman-centered care that today's society demands against disrespect, mistreatment and other behaviors already categorized in many parts of Latin America as obstetric violence. Health professionals involved in the care of pregnant women and childbirth cannot continue to ignore the growing scientific evidence regarding the importance of the physiological evolution of labor, the emotional and psychological aspects of women during the birth of their babies and the long-term effects on mothers and newborns of the perinatal event and early parenting. We can conclude on the importance of educating future parents, of implementing actions during cesarean section such as skin-to-skin contact and monitoring of the first breastfeeding and of professional follow-up to support breastfeeding difficulties after birth. These various factors were of great importance for women with complicated pregnancies in support of achieving more exclusive and prolonged breastfeeding, even for more than 6 months. We must promote that more and more nurses, physicians, and personnel in contact with mothers and newborns acquire adequate knowledge about breastfeeding in order to rescue this valuable resource with short and long term benefits in children's health beyond what we still do not fully understand.

## Data availability statement

The raw data supporting the conclusions of this article will be made available by the authors, without undue reservation.

## Ethics statement

The studies involving human participants were reviewed and approved by Comité de Ética del Hospital General del Estado de Sonora. The patients/participants provided their written informed consent to participate in this study.

## Author contributions

JZ-S contributed with the conceptualization, design of this study, acquisition of data, ran formal analysis, and organized transcriptions. CT-F contributed by supervising this study, its methodological tasks, and data interpretation. LH-R made substantial contributions by editing and revising the manuscript critically for important intellectual content. JZ-S and LH-R provided the writing of the original draft. All authors contributed to manuscript revision, read, and approved the submitted version.

## Conflict of interest

The authors declare that the research was conducted in the absence of any commercial or financial relationships that could be construed as a potential conflict of interest.

## Publisher's note

All claims expressed in this article are solely those of the authors and do not necessarily represent those of their affiliated organizations, or those of the publisher, the editors and the reviewers. Any product that may be evaluated in this article, or claim that may be made by its manufacturer, is not guaranteed or endorsed by the publisher.
