# Supplementary material for: Pro-lactation cesarean section: Immediate skin-to-skin contact and its influence on prolonged breastfeeding
Source: Front Sociol. 2022 Sep 27;7:908811. doi: 10.3389/fsoc.2022.908811 (PMC9551215; doi:10.3389/fsoc.2022.908811)
Supplement: Supplementary file 1 [file Data_Sheet_1.PDF]

Supplementary Material

Skin to skin Contact Procedure

1.The procedure is explained to the mother and her partner or companion, and their consent is requested.
2. The benefits of breastfeeding are explained to mothers
3. Coordination with the procedure anesthesiologist to perform anesthesia with the minimum medication sufficient to keep the mother calm but alert during the procedure and to monitor the mother in such a way as to leave the chest free (in shoulders or on the back).
4. Asepsis and antisepsis of the abominous genital region is performed preferably with chlorhexidine to avoid prolonged contact of the baby with iodine. If it is necessary to use iodine, excess is removed and its placement is limited to the ribcage only.
5. Fold of the end of the head field, left with the fold to the sterile area to use as a base when placing the newborn with the intact cord on its mother.
6. The mother receives a brief explanation and is reminded not to move her arms until she is told that she can hold her baby and that she must not touch the sterile fields beforehand.
7. When the baby is born, the surgical field is lowered, trying to fold the sterile cephalic field in the form of a pleated curtain so that the mother, if she wishes, can see the birth of her son/daughter.
8. The baby is gently and slowly extracted through the abdomen, allowing self-resuscitation simulating vaginal birth to favor the expulsion of pulmonary fluid as well as the physiological transition of the newborn.
9. The newborn is dried to avoid heat loss, avoiding drying of the hands to favor sniffing and suckling on them.
10. The baby is passed to the person in charge of his care who also wears sterile clothes so that he/she can be placed on the mother's torso while waiting for the timely cutting of the umbilical cord, while at the same time evaluating the baby on his/her mother's chest.
11. The newborn must be naked, face down (prone) on the mother's chest, it can be in the middle of both breasts or obliquely, with the head on one side and covered with a warm blanket.
12. Oximeter and pressure cuff will be removed to make it easier for the mother to hug her baby.
13. The cord will be clamped in a timely manner, taking into account the physiological adaptation of the newborn, or prolonging the cut for at least 1

to 3 minutes and even until it stops beating if the mother's conditions allow it.
14. The surgical fields are raised once the cord has been clamped, ensuring that the area that was in contact with the baby remains on the non-sterile maternal side.
15. Skin-to-skin contact will be maintained throughout the duration of the intervention, if possible, or done with the father if the mother does not feel well or is unsure of taking charge.
16. The mother is encouraged to initiate breast feeding if the newborn initiates hunger and foraging behaviors.
17. If necessary, the newborn is cared for in a radiant heat cradle within the same operating room and is returned to the mother as soon as possible to continue with skin-to-skin contact
18. Once the intervention is over, continuity of skin-to-skin contact is favored in the recovery area, including the mother's partner, helping to keep the new family together.
19. The first feeding to the mother's breast is favored, guided, and supervised within the first hour of life of the newborn, either in the operating room or in post-operative recovery.
20. Once the new mother has recovered, they go together to her room to continue in joined accommodation or “Rooming in”

Supplementary Table 1. Detailed step-by-step description of the skin-to-skin contact procedure during cesarean section delivery.

Dimension	*Themes*
**Maternal socio-demographic factors**	**Mother's Education, Preparation for birth, Intention to breastfeed, Birth Complications**
Example of response obtained	"I had planned a water birth, but the circumstances did not work out because the labor since my water broke was very long and tiring, I did not sleep for 24 hours and the baby's heart began to accelerate, that was when I started to make decisions that I had not planned, first the epidural to calm me down and then after a few hours came the cesarean section for not having gone down."<br><br>"Knowing all the benefits of skin-to-skin was something I was looking for in my delivery and we had it. I wouldn't change a thing about the moment of my daughter's birth."
**Maternal care and birth services**	**Management of cesarean section, Immediate skin to skin contact after delivery, Information about lactation, Support from healthcare professionals and Spatial proximity ("rooming in")**
Example of response obtained	"It was a unique and very emotional moment, they put him on my chest, and we made skin to skin contact, the most impressive thing was that at that moment my baby looked for my breast and started to suck."<br><br>"On the day of the birth the entire medical team made me feel safe and calm, from the nurses to the anesthesiologist and the doctors were respectful and kind, making me feel like the protagonist of the birth."<br><br>"Even though I was under anesthesia and in surgery, feeling my baby skin to skin gave me the gift of relaxation to my body. When I left the room my baby stayed with me, she did not go to the nursery. For two hours I was able to establish a special connection with her and she fell asleep on my chest."<br><br>"The first night we were in the same room with my baby, my husband and I, since the doctor told us that it is very important for the baby to bond from the very first moment."
**Successful lactation**	**Early Lactation, Exclusive Lactation, Prolonged lactation, Breastfeeding Duration, Level of satisfaction with their delivery and lactation process.**
Example of response obtained	"I was able to feed my baby in the operating room, it was the best, breastfeeding was something I was very excited about, and we did it."<br><br>"In my case, when I breastfed him and he quickly looked for it and latched on, for me it is nice because you think about the labor, the hours that lasted were important, they were useful, and he quickly looked for it even though it was not natural and physically you do not see the milk, but you know that it is there, and he did not take formula."

Supplementary Table 2. Example of responses obtained for each dimension.

Instrument	*Sample Question*
**Survey**	If you had any complications during pregnancy or birth of your baby, what were they?<br><br>When did you first feed your baby formula or any food or liquid other than breast milk?<br><br>Do you think you have managed to exclusively breastfeed your baby for the first six months of life?<br><br>Are you satisfied with your baby's birth method?<br><br>Was the birth of your baby as you expected?
**Interview**	Describe your personal experience with the birth of your baby<br><br>Were you in skin-to-skin contact with your baby for at least 30 minutes during the first two hours of life? Please talk about that experience.<br><br>Were you with your baby from birth to discharge in the same room? How did you feel about it?<br><br>Do you consider that you have received help and useful information after the birth of your baby to initiate breastfeeding?<br><br>How long did you manage to breastfeed your baby?<br><br>How do you feel about feeding your baby for the first two years of his life?

Supplementary Table 3. Sample questions from the applied survey and interview.
